# Acute uropathogen-related cowperitis with sepsis: case report and literature review

**DOI:** 10.1016/j.eucr.2023.102566

**Published:** 2023-09-13

**Authors:** Giuseppe Maiolino, Andrea Boni, Michele Del Zingaro, Miriam Russo, Ettore Mearini

**Affiliations:** Department of Surgical and Biomedical Sciences, Division of Urology Clinic, University of Perugia, Perugia, 06129, Italy

**Keywords:** Cowperitis, Sepsis, Case report, MAGI, Perineal ultrasound

## Abstract

Acute cowperitis, which was previously known as a common complication of sexually transmitted infections (STIs), is now commonly associated with bacterial urinary tract infections, particularly *Escherichia coli*. Patients often have a history of STIs, and the symptoms resemble other male accessory gland infections (MAGIs). Recent cases associated with sepsis have been managed with percutaneous drainage and/or surgery. We present a case of acute cowperitis with sepsis and an abscess in the right small gland. The diagnosis was made using transperineal ultrasound, and the patient was successfully treated only with a long-term antibiotic therapy.

## Highlights

• A cowperitis is an infection, either acute or chronic of the bulbourethral glands.
• Cowperitis were known as a complication of Sexually Transmitted Infections.
• Actually, AC are associated with uropathogens (e.g. *Escherichia coli*).
• Recent cases have been treated with percutaneous drainage and/or surgery.
• We report a case successfully treated only with a long-term antibiotic therapy.

## 1 Introduction

Cowperitis is an infection, either acute or chronic of the bulbourethral glands.^1^ Recently, two cases of acute cowperitis with a similar pattern history, clinical presentation and evolution were reported in the literature.^2^, ^3^, ^4^ We present a case caused by Escherichia Coli and complicated by sepsis, successfully treated with a conservative management without long-term complications.

## 2 Case presentation

We followed the CARE checklist for this case report. A 50-year-old man presented to our department in January 2021 with fever (>38 °C), low blood pressure, tachycardia, perineal pain, and storage lower urinary tract symptoms. Blood tests revealed leukocytosis, elevated hs-C-Reactive Protein and procalcitonin levels. Abdomen ultrasound and chest x-ray showed no pathological findings.

The patient's general medical history resulted negative for pathological findings, even his sexual history: the patient reported no previous SITs and he also stated that he regularly used condoms during sexual intercourse with his regular female partner.

**Fig. 1:**
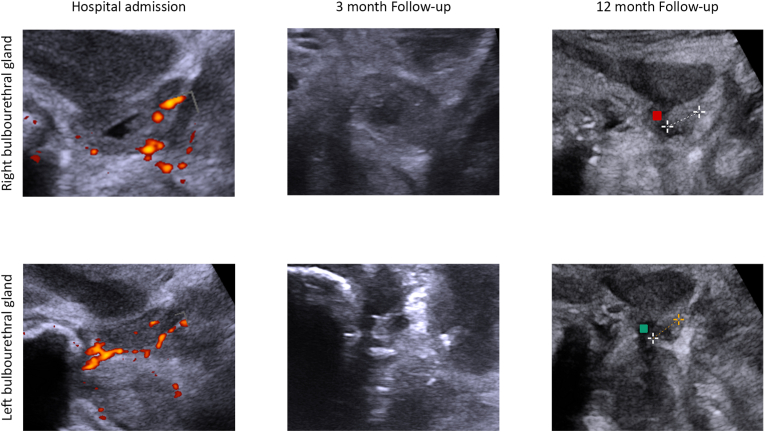
Perineal ultrasound findings at hospital admission, 3 month-follow up and 12-month follow-up Hospital admission: the right bulbouretheral gland appeared distended with a hypoechoic area in the center and showed abnormal increased intraparenchymal vascularization at Power-Doppler. The left bulbourethral gland appeared isoechoic and enlarged with evidence of increased vascularization around the gland. **3-months follow-up**: the right bulbourethral gland appeared equal in dimensions but with reduced hypoechoic area. The left bulbourethral gland appeared smaller and isoechoic. **12-month:** normal echotexture and size of both glands.

Upon urological examination, patient's prostate, penis and testis were found to be normal. However, a bi-digital rectal examination revealed a swelling in the right perineal urogenital diaphragm. Subsequently, a perineal ultrasound was performed, and the ultrasound findings are depicted in Fig. 1.

Urethral swab, urine and blood cultures were taken. Pain killers and empirical antibiotic treatment were started (Piperacillin/Tazobactam 4 gr/0,5 gr every 6 hours IV) and a urinary catheter was placed. Urine and blood culture resulted positive for non-ESBL E.coli and urethral swab with PCR (Polymerase Chain Reaction) negative for *Neisseria gonorrhoea*, *Chlamydia trachomatis* and *Trichomonas vaginalis.*

After 2 days, a good clinical response (defervescence) was observed, but at 5 days from hospital admission, the patient presented new daily fever spikes (>38 °C) with generalized malaise and a new rise of hs-CRP and procalcitonin levels. New blood cultures still resulted positive for non-ESBL E.Coli and the antibiotic regimen was changed to Teicoplanin 500 mg every 24 hours IV plus Ertapenem 1 gr every 24 hours IV. A transthoracic echocardiography ruled out vegetations of infective endocarditis.

A total of 21 days of IV antibiotic therapy led to a good and stable clinical and biochemical response (normal levels of hs-CRP and procalcitonin). At hospital discharge, a broad-spectrum antibiotic therapy was given for another 6 weeks.

At 3-, 6-, 12- and 24-month outpatient visits, the patient was asymptomatic for LUTS with normal blood values (normal white cell count and hs-CRP and procalcitonin levels). Perineal ultrasound findings at 3-month and 12-month follow-up were compared to those of admission and showed in Fig. 1.

Patient expressed high satisfaction with the treatment received and provided written informed consent for the publication of this case report.

## 3 Discussion

The clinical presentation of acute cowperitis is similar to other Male Accessory Gland Infections (MAGIs), characterized by severe perineal pain, voiding urinary symptoms, fever and malaise.^1^ Some cases may also experience acute urinary retention.^5^ Abnormal findings during digital rectal examination include an enlarged and thickened urogenital diaphragm which can be felt when grasped between thumb and forefinger. For diagnosis, urine culture, urethral swab with PCR tests and blood cultures are the primary microbiological tests used.

While rare case of cowperitis have been described in the literature such as stone, male genital tuberculosis or infected syringocele, historically, STIs such as *Neisseria gonorrhoea* and *Chlamydia trachomatis* were the main principal causes*.* Schischow and Smirnov reported a prevalence of 12.2% of Cowperitis in a historical series of 200 cases of *Neisseria gonorrhoea* infection.^1^

Currently acute Cowperitis is typically associated with bacteria commonly involved in urinary tract infections, such as *Escherichia coli* and *Pseudomonas aeruginosa*, although a history or concomitant infection of STIs-pathogens is frequently observed. Recent cases reported in the literature suggest the possibility of a two-step infection process, wherein an initial STI is followed by suprainfection of uropathogens.

Pepe et al. reported an acute cowperitis caused by Escherichia Coli, *Chlamydia trachomatis* and Pseudomonas Aeruginosa. The patient was treated with ultrasound-guided aspiration and two months later, he underwent transperineal surgical asportation for a recurrence of the abscess. However, at one month after the surgery, the patient was readmitted due to the development of a urinary fistula between the site of the previous Cowper's gland excision and the bulbar urethra. The patient was treated with suprapubic catheterization and antibiotic therapy.^2^^,^^3^

Kadyrov Z et al., reported a patient with a history of *Chlamydia trachomatis* and Ureaplasma urealyticum infections and a percutaneous bulbourethral abscess drainage six months prior to the hospital admission. The patient presented in septic shock state and underwent rapid hemodynamic stabilization in the intensive care unit before undergoing surgical treatment. The last urine culture of patients had showed an *E. coli* and S. saprophytes growth; and intraoperative urine culture detected *E. coli* and *K. pneumoniae*.^4^

These cases highlighted the presence of a concomitant or previous *Chlamydia trachomatis* infection, while in our case there were no findings related to STIs. However, an undiagnosed past STI cannot be excluded in our patient. The features of the three cases have been summarized in Table 1.

**Table 1:** Acute cowperitis uropathogen-related reported in literature.

	Pepe P. et al., 2017, 2019^2^^,^^3^	Kadyrov Z et al., 2021^4^	Maiolino et al. (current case)
**Age**	63	63	50
**History findings**	TURP A recent urine culture positive for E. Coli infection and *Chlamydia trachomatis*	Repeatedly treated for infections caused by *Chlamydia trachomatis* and Ureaplasma urealyticum Aspiration of a bulbourethral abscess performed 6 months before	Negative
**Microbiologic findings**	Blood culture: *E. coli* Culture from percutaneous drainage of Cowper's abscess: Pseudomonas Aeruginosa	Urine culture: *Escherichia coli*; *Klebsiella pneumoniae*	Urine culture and blood culture: non-ESBL E.Coli
**Clinical presentation**	Sepsis, perineal pain, storage urinary symptoms	Septic shock, pain and enlargement of the perineum and the scrotum, storage urinary symptoms	Sepsis, painful perineal swelling, storage urinary symptoms
**Imaging**	Transrectal and perineal ultrasound; Pelvic MRI	Transrectal ultrasound; Pelvic MRI	Perineal ultrasound
**Treatments**	Antibiotic therapy and transperineal ultrasound-guided aspiration of Cowper's abscess, followed by suprapubic urinary catheter	Surgical drainage of bulbourethral abscess with a scrotal-perineal approach	21-days of IV antibiotic therapy +6 week of oral broad-spectrum antibiotic therapy
**Follow-up**	Recurrence two months later, treated with transperineal surgical asportation. At 1 month after surgery, urinary fistula between the site of the previous Cowper's gland excision and bulbar urethra, treated with suprapubic catheterization and antibiotic therapy	Fistula between the urethra and Cowper's gland, treated with suprapubic urinary catheter removed after six months	At 24 months patients resulted asymptomatic

When uropathogens are involved in acute cowperitis, common features could be summarized as follows: 1) Sepsis can be the initial clinical manifestation; 2) There is a tendency for acute abscess formation 3) Polymicrobial agents are often involved 4) Poor response to antibiotic therapy and percutaneous drainage; 5) Recurrence after a long period of state of well-being.

Treatments options reported are antibiotic therapy, placement of a suprapubic catheter, ultrasound-guided aspiration and *trans*-perineal surgical asportation. In our case, since a small defined acute abscess formation was present, the decision was made to avoid percutaneous drainage and instead use a long course of antibiotic therapy. No complications were noted at 12 and 24-month of follow-up. Based on our experience, a longer broad-spectrum antibiotic-therapy is probably necessary for acute cowperitis compared to other MAGIs therapies reported in guidelines.

## 4 Conclusion

Acute Cowperitis associated with uropathogens is a rare but serious condition. In cases where a small and defined abscess is present, a longer course of antibiotic therapy appears to be necessary compared to percutaneous drainage to prevent early and late potential complications.

## Consent

Patient provided a written informed consent to publish this case report.

## Funding

This research did not receive any specific grant from funding agencies in the public, commercial, or not-for-profit sectors.

## Author contributions

Conceptualization, M.G., B.A; methodology, M.G., M.R; writing—original draft preparation, G.M. B.A.; writing—review and editing M.R., M.D.Z., M.E.; supervision, M.E.; project administration G.M.

## Declaration of competing interest

The authors declare no conflict of interest.

## References

[1] Harkness A.H. (1937). Infections of the bulbo-urethral glands of cowper. Br J Vener Dis. Apr.

[2] Pepe P., Pepe L., Bonaccorsi A., Panella P., Pennisi M. (Oct 2 2019). A complicated case of recurrent Cowper's gland abscess. Arch Ital Urol Androl.

[3] Pietro P., Ludovica P., Astrid B., Paolo P., Michele P. (2017). Sepsis secondary to Cowper's gland abscess. Urol Case Rep. Nov.

[4] Kadyrov Z., Stepanov V., Aldyrakov E., Ramishvili S. (2021). Cowper's glands abscess with spreading to adjacent organs and tissues with development of septic shock: an extremely rare case. Res Rep Urol.

[5] Chughtai B., Sawas A., O'Malley R.L., Naik R.R., Ali Khan S., Pentyala S. (2005). A neglected gland: a review of Cowper's gland. Int J Androl. Apr.

